# Extrapulmonary tuberculosis and HIV

**DOI:** 10.4103/0970-2113.76312

**Published:** 2011

**Authors:** Syed Ahmed Zaki

**Affiliations:** *Department of Pediatrics, Lokmanya Tilak Municipal General Hospital, Sion, Mumbai 400022, India E-mail: drzakisyed@gmail.com*

Sir,

I read with interest the recent case report on “Parotid tuberculosis” by Garg *et al*,[1] and have the following comments to offer:

1. The statement made by the authors “Since 1893, only about one hundred cases of parotid gland tuberculosis have been reported in the literature” is incorrect. The reference cited for the statement is from an article published in 1996 which mentions the total number of cases described till that year. Ideally, the authors should have done an extensive literature search on the number of published cases of parotid gland tuberculosis till date. A search on PubMed with the keywords “tuberculosis” AND “parotid gland” revealed more than 40 cases of parotid gland tuberculosis published after 1996.
2. HIV testing was not done in the case described by the authors. HIV positive patients are more likely to present with extrapulmonary or sputum smear-negative tuberculosis as compared to HIV negative patients.[2] Extrapulmonary tuberculosis has been accepted as an AIDS-defining criterion. The WHO clinical staging of HIV/AIDS is used in many countries to determine eligibility for antiretroviral therapy, particularly in settings in which CD4 testing is not available. Presence of extrapulmonary tuberculosis puts a patient in stage 4 which is an indication for starting ART.[3] Furthermore, HIV-infected tuberculosis patients are a priority for epidemiologic investigation because these persons are more likely to have HIV-infected contacts than are seronegative tuberculosis patients. Also, irrespective of epidemic setting, WHO recommends HIV testing for patients of all ages in whom tuberculosis is suspected or already confirmed.[4] Through this letter, I would like to re-emphasize our readers that HIV testing should be done in cases of tuberculosis, especially those with extrapulmonary manifestations.
